# Erythrodermic crusted scabies—a diagnostic pitfall and mini-review: a case report

**DOI:** 10.3389/fmed.2026.1862964

**Published:** 2026-07-13

**Authors:** Juris Podoļanskis, Kristīne Nevidovska, Artūrs Kaļva, Lāsma Kalnbērza

**Affiliations:** 1 Department of HIV/AIDS (Dermatology-Venerology), Riga East University Hospital, Latvian Centre of Infectious Diseases, Riga, Latvia; 2 Department of Pathology, Riga East University Hospital, Centre of Pathology, Riga, Latvia; 3 Institute of Public Health, Riga Stradiņš University, Riga, Latvia

**Keywords:** case report, crusted scabies, erythroderma, erythrodermic crusted scabies, ivermectin

## Abstract

**Introduction:**

Erythroderma is a severe dermatologic condition with a broad differential diagnosis encompassing inflammatory, drug-induced, neoplastic, and infectious etiologies. Scabies is the most common neglected tropical disease (NTD) and can manifest in two common forms: classic and crusted types. Although rare, crusted scabies is an important and frequently underrecognized cause of erythroderma (accounting for <0.5% of cases) that poses diagnostic challenges due to its clinical overlap with other dermatoses associated with erythroderma.

**Case description and review:**

We report the case of a 70-year-old man with a history of psoriasis who developed generalized erythroderma with pruritus and crusted, hyperkeratotic plaques on the face, scalp, and trunk. Skin biopsy demonstrated psoriasiform changes without detectable mites, whereas skin scrapings confirmed the presence of *Sarcoptes scabiei*, establishing the diagnosis of crusted scabies (erythrodermic crusted scabies). Differential diagnoses included psoriasis, adverse drug reaction, and seborrheic dermatitis. Treatment with oral ivermectin and topical scabicidal therapy resulted in marked clinical improvement. A review of the literature identified 16 reported cases of erythroderma secondary to scabies and highlighted the frequent misdiagnosis of this condition due to overlapping clinical and histopathological features, potentially delaying appropriate treatment and increasing the risk of complications.

**Conclusion:**

This case highlights the importance of considering crusted scabies in patients presenting with erythroderma, pruritus, and hyperkeratotic or scaly lesions and supports the integration of clinical assessment with parasitological examination, particularly skin scrapings, to reduce the risk of misdiagnosis and facilitate timely management. This is particularly relevant in primary care and general practice settings, where initial management decisions are often made. Knowledge of various scabies manifestations, especially its erythrodermic presentation, could further improve disease management and control, particularly in endemic and resource-limited areas.

## 1 Introduction

Erythroderma, defined as diffuse erythema and scaling involving most of the body surface area, may represent the manifestation of various underlying diseases (1). Its clinical presentation is often challenging and may be difficult to interpret with respect to the underlying cause. The most common etiologies include psoriasis, drug-induced reactions, atopic dermatitis, cutaneous T-cell lymphoma, and contact dermatitis (1). Up to 30% of cases may remain idiopathic (1). Although less common, infestations such as scabies, including the crusted scabies variant, are also recognized causes of erythroderma (2). While some patients may tolerate the physiological disturbances associated with erythroderma, elderly and frail individuals with multiple comorbidities are at increased risk of life-threatening complications, including sepsis, high-output cardiac failure, electrolyte and metabolic disturbances, and end-organ damage (1).

Crusted scabies (previously known as “Norwegian scabies”) is a rare and severe form of scabies caused by the ectoparasite *Sarcoptes scabiei* var. *hominis* (3, 4). It is characterized by intense pruritus and thick, crusted, hyperkeratotic lesions resulting from massive mite proliferation and an altered host immune response, whereas classic scabies typically lacks marked hyperkeratosis (3, 4). Although hyperkeratosis is a hallmark of crusted scabies, erythrodermic presentations are uncommon, which may contribute to diagnostic challenges because the condition can mimic other erythroderma-initiating dermatoses (2). This diagnostic complexity is especially relevant in primary care settings, where initial evaluation is often performed.

Scabies is a global parasitic infestation that is most prevalent in low-income, densely populated, and tropical regions (5). In recent years, sporadic cases and outbreaks have also increased in high-income countries, particularly in institutional settings such as hospitals, long-term care facilities, and prisons (6). Similar trends have been observed across Europe, with rising incidence reported in Germany, Norway, France, and Croatia (5). In Germany, the number of reported scabies cases increased approximately ninefold between 2009 and 2018, predominantly among adolescents and young adults (6). Similarly, the Netherlands experienced a threefold increase in cases over a decade, largely attributed to institutional outbreaks (6).

In this study, we report the case of a 70-year-old man with a history of psoriasis who developed erythroderma associated with crusted scabies. In addition, we present a review of the literature on erythroderma secondary to scabies, summarizing reported cases and emphasizing the potential for misdiagnosis and its therapeutic implications. This case and review highlight the risk of misdiagnosis. Erythroderma is often misdiagnosed as an inflammatory dermatosis, leading to inappropriate immunosuppressive therapy. In undiagnosed classic or crusted scabies, such treatment may worsen disease progression, including the development of erythroderma, and delay proper intervention. Effective management requires targeted antiparasitic therapy rather than immunosuppression.

## 2 Materials and methods

A literature review was conducted to identify previously reported cases of erythrodermic crusted scabies. The search was performed in the Scopus database using the term “erythrodermic crusted scabies” for articles published between 2004 and 2023, yielding 13 results. PubMed was searched using “erythroderma AND crusted scabies,” as the term “erythrodermic crusted scabies” yielded only one result; this search (2006–2026) identified eight articles. In total, 21 articles were retrieved. Titles, abstracts, and full texts were screened to identify relevant case reports. Accessible English-language articles were included, and duplicates were excluded. After applying the inclusion criteria, 16 studies remained for the final review. Key data from these cases—including authors, publication year, patient demographics, initial diagnoses, risk factors (e.g., corticosteroid use and immune status), treatment, and outcomes—are summarized in Table 1.

**Table 1:** Reported cases of erythrodermic crusted scabies identified in the literature review (*n* = 16).

Authors (year)	Age/sex	Initial diagnosis	Risk factors/immune status	Diagnostic test	Treatment	Outcome
Binić et al. (2009) (11)	62/F	Erythroderma in relation to hypothyroidism	Elderly; systemic and topical corticosteroids	Skin scrapings	Topical 1% lindane; topical 25% benzyl benzoate; topical 10% sulfur ointment	Complete resolution
Garlatti et al. (2015) (12)	72/F	Primary hypereosinophilic syndrome	Elderly; systemic corticosteroids	Dermoscopy; skin scrapings; histology	Not reported	Not reported
Cartron et al. (2020) (13)	Not reported/F	Erythrodermic psoriasis	Elderly; systemic corticosteroids	Histology	Topical 5% permethrin lotion, oral ivermectin 200 μg/kg on days 1, 2, 8, 9, and 15	Clinical improvement; Death due to sepsis
Chen et al. (2024) (14)	74/M	Eczema or psoriasis	Elderly; topical corticosteroids; Parkinson’s disease; hemiparesis	Skin scrapings; histology	Topical 20% sulfur cream	Clinical improvement; Death due to sepsis
Tolkachjov et al. (2018) (15)	90/F	Recalcitrant contact dermatitis	Elderly; topical treatments; systemic corticosteroids; mycophenolate mofetil	Skin scrapings; histology	Oral ivermectin 200 μg/kg on days 1, 2, 8, 9, and 15; topical 5% permethrin; oral prednisone slowly tapered	Not reported
Devi and Hazarika (2021) (16)	45/M	Erythrodermic psoriasis	Self-prescribed steroids (iatrogenic Cushing’s disease)	Skin scrapings	Oral ivermectin 200 μg/kg on days 1, 2, 8, 9, and 15; topical 5% permethrin cream; physiologic dose of prednisolone for iatrogenic Cushing’s disease	Resolution of hyperkeratotic plaques
Dragoš et al. (2004) (17)	8/F	Juvenile dermatomyositis (confirmed earlier) with Gottron papules	Systemic and topical corticosteroids (for juvenile dermatomyositis)	Skin scrapings	Bland emollients and systemic antibiotic; topical 6% precipitated sulfur ointment	Complete resolution
Fonseca et al. (2013) (18)	3/F	Erythrodermic psoriasis and onychomycosis	Down syndrome; topical corticosteroids; cyclosporine	Skin scrapings	Oral ivermectin 200 μg/kg once weekly for 4 weeks; topical 5% permethrin cream	Clinical improvement in nail dystrophy and no recurrence of keratoderma
Kulkarni et al. (2016) (19)	71/M	Psoriasis, eczema, and cutaneous lymphoma in differential diagnoses	HIV	Skin scrapings; histology	Parenteral ceftriaxone; oral ivermectin 12 mg stat dose (repeated after 1 week); oral hydroxyzine; topical 5% permethrin cream; (urea 10% + lactic acid 10% + propylene glycol 10% + liquid paraffin 10%) cream; previous ART and AKT continued	Clinical improvement
Olamiju et al. (2023) (20)	84/M	Not reported. Patient was treated for GPA	Azathioprine (for GPA) induced myelosupression; oral corticosteroids	Histology; skin scrapings	Topical 5% permethrin cream; oral ivermectin 200 μg/kg on days 1 and 10	No recurrence of scabies
Paparizos et al. (2019) (21)	55/M	Not reported	HIV (discontinuation of antiretroviral therapy), unsanitary conditions, and malnourishment	Skin scraping	Topical 25% benzyl benzoate; topical 5% urea/corticosteroid cream; oral antihistamines; long-acting betamethasone intramuscular injection; initiation of ART	Complete resolution
Piana et al. (2015) (22)	75/M	Erythrodermic psoriasis or adverse drug reaction	Elderly; systemic corticosteroids	Histology	Topical 5% permethrin	Not reported
Ren et al. (2024) (23)	78/F	“Senile pruritus, eczema, and hypereosinophilic dermatitis”	Elderly; topical and systemic corticosteroids	Dermoscopy; skin scrapings	Ivermectin 200 μg/kg once weekly for 4 weeks; sulfur ointment	Resolution
Shrestha and Bischof (2021) (24)	73/M	Delay in diagnosis; erythroderma in relation to hypereosinophilia	Elderly; systemic corticosteroids	Skin scrapings (negative); histology (positive)	Topical permethrin; oral ivermectin; oral prednisone with gradual taper for scabies-induced hypereosinophilic syndrome	Significant improvement
Talty et al. (2022) (25)	80/F	Crusted scabies considered in differential diagnoses along with atopic dermatitis, CTCL, psoriasis, and autoimmune connective tissue disease	Elderly; topical and systemic corticosteroids; dupilumab (?)	Histology	Oral ivermectin 200 μg/kg on days 1 and 10; topical 5% permethrin	Clinical improvement
Widaty et al. (2021) (26)	32/M	Eczema	Topical and systemic corticosteroids	Skin scrapings; dermoscopy	Topical 5% permethrin; oral clindamycin; oral cetirizine; normal saline wet dressing on crusts and fissures; tapering of oral corticosteroid	Significant improvement

## 3 Case description

**Figure 1:**
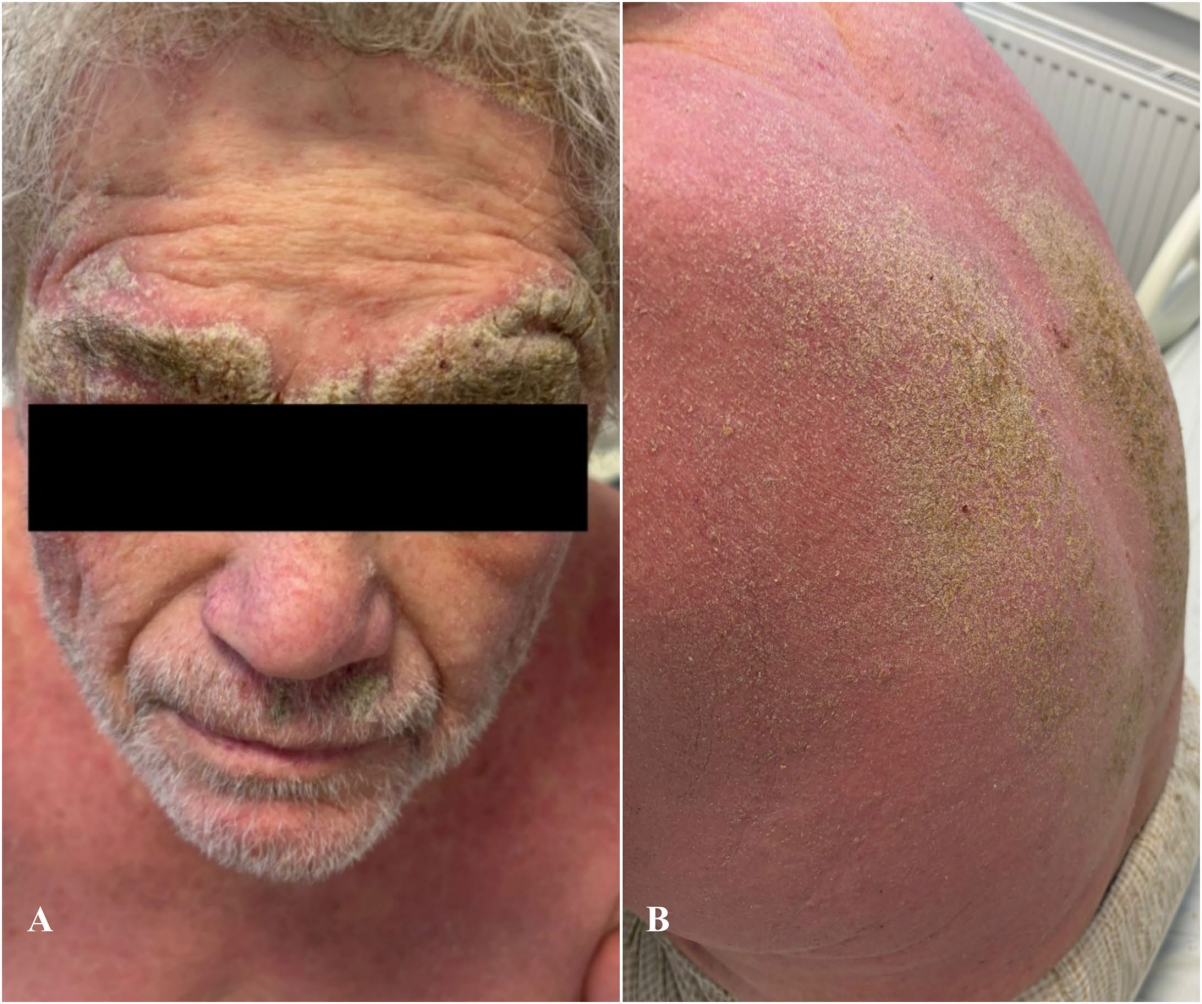
Clinical presentation of the patient’s condition showing generalized erythema (erythroderma) and hyperkeratotic, scaly plaques along the hairline, eyebrow, and beard region **(A)**; some papules are also visible on the forehead. Similar scaly plaques were also observed on the patient’s back **(B)**.

A 70-year-old man presented to the Department of Dermatology and Venereology in January 2026 with a several-month history of pruritus and widespread erythematous skin lesions. The condition began in July 2025 with pruritic papular lesions, which were initially misdiagnosed as an allergic reaction. Despite intermittent treatment with oral antihistamines and topical corticosteroids, accompanied by only short-term remissions, the patient’s condition continued to deteriorate and ultimately progressed to generalized erythroderma. The patient stated that he had never experienced a similar episode of widespread erythema before. The patient had no history of close contact with individuals with similar symptoms or institutional exposure. His medical history was notable for psoriasis with intermittent involvement of the elbows, which was treated with topical corticosteroids applied intermittently to the affected areas. On examination, erythroderma involved most of the trunk and extremities and was associated with moderate-to-severe crusted yellow hyperkeratotic plaques on the back, face (notably the eyebrow region), and scalp (Figure 1). In addition, lichenification and excoriations were observed on the arms. His medical history included diabetes, benign prostatic hyperplasia, hyperlipidemia, and cardiovascular disease. His medications included allopurinol, rosuvastatin, gliquidone, acetylsalicylic acid, dutasteride/tamsulosin, metformin/empagliflozin, and metoprolol. All reported medications had been initiated several years before the onset of erythroderma; for example, acetylsalicylic acid had been started approximately 20 years earlier for the prevention of blood clots. Although the patient was unable to recall the exact initiation dates for all medications, no recent changes were identified in his therapy.

Laboratory tests showed mild leukocytosis (11.62 × 103/μL), significant eosinophilia (1.78 × 103/μL), slight normocytic anemia (Hb 12.9 g/dL), and mildly elevated C-reactive protein (11.7 mg/L). HbA1c was 6.05%, and HIV serology was negative. The differential diagnoses included erythrodermic psoriasis, initially considered because of the patient’s known history of psoriasis; crusted scabies, which may present with similar clinical features; erythrodermic seborrheic dermatitis, given the involvement of the scalp and face with hyperkeratotic lesions; and an adverse drug reaction, as certain medications, including β-blockers (e.g., metoprolol) and non-steroidal anti-inflammatory drugs (NSAIDs; e.g., acetylsalicylic acid), have been reported to trigger or exacerbate psoriasis.

**Figure 2:**
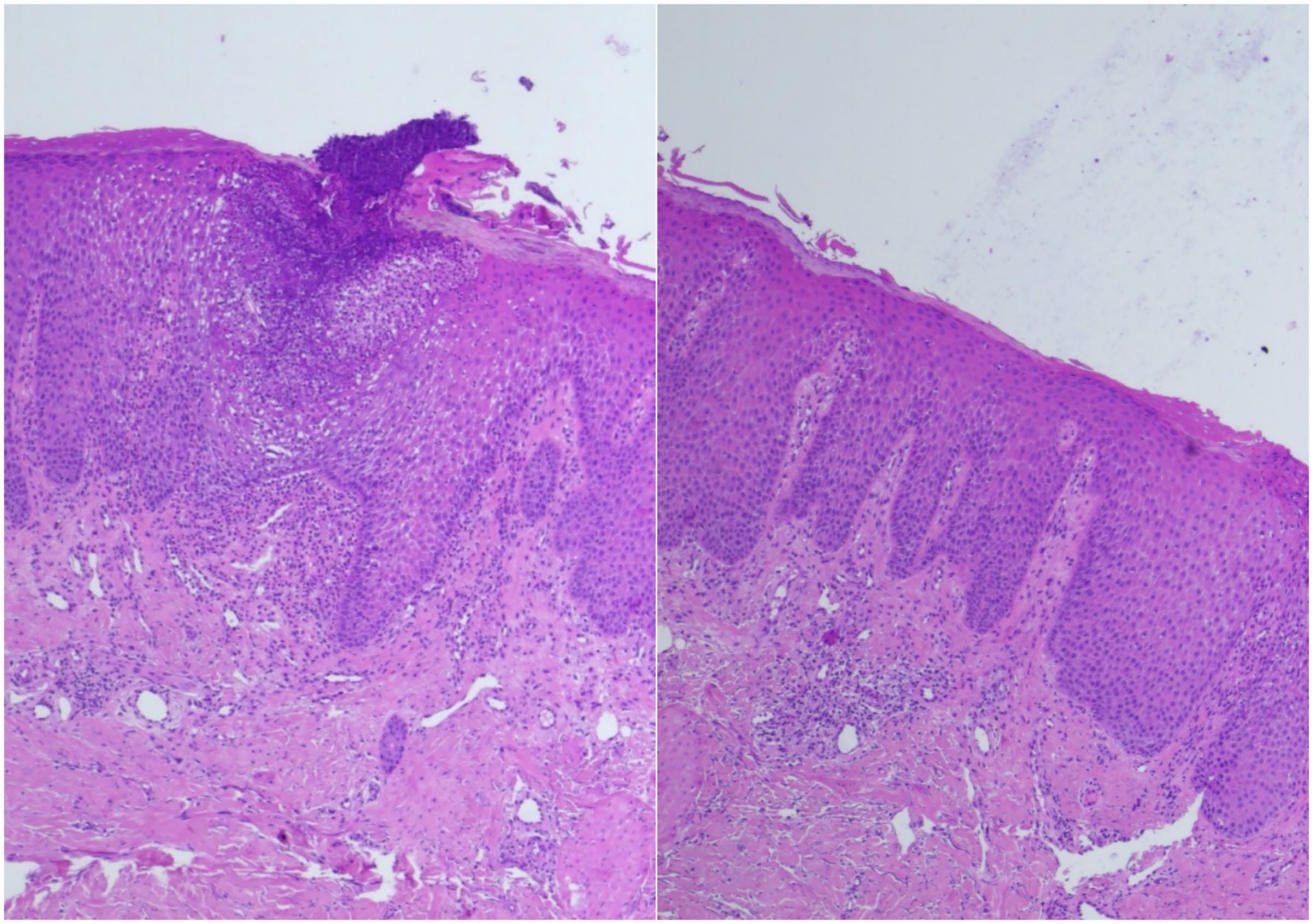
Histopathology of a skin biopsy (H&E stain, 10 × magnification) showed a thick hyperkeratotic stratum with areas of parakeratosis containing small plasma coagula and focal leukocyte collections. The epidermis showed significant irregular acanthosis, hyperplasia, and focal spongiosis, with neutrophilic collections forming pustules in the upper layers. The papillary dermis contained perivascular neutrophils and occasional lymphocytes, with some neutrophils present within vessel lumina. The deeper dermis was free of significant inflammation. No structures suggestive of Sarcoptes scabiei were identified. Histopathology demonstrated psoriasiform changes suggestive of psoriasis.

Dermoscopy was performed and revealed findings suggestive of scabies, including burrows and the delta-wing sign. Due to the atypical clinical presentation, a skin scraping examination was subsequently performed. Microscopic examination of skin scrapings from the face, back, and arms revealed numerous mites and eggs, confirming scabies. A punch biopsy was performed on a crusted lesion on the lateral aspect of the back. Histopathological examination revealed non-specific psoriasiform changes without identifiable *Sarcoptes scabiei* (Figure 2). The diagnosis was also supported by the 2020 International Alliance for the Control of Scabies consensus criteria for the diagnosis of scabies. In this case, criteria A1 (mites, eggs, or feces identified on light microscopy of skin scrapings) and A3 (mites visualized on dermoscopy) were fulfilled.

**Figure 3:**
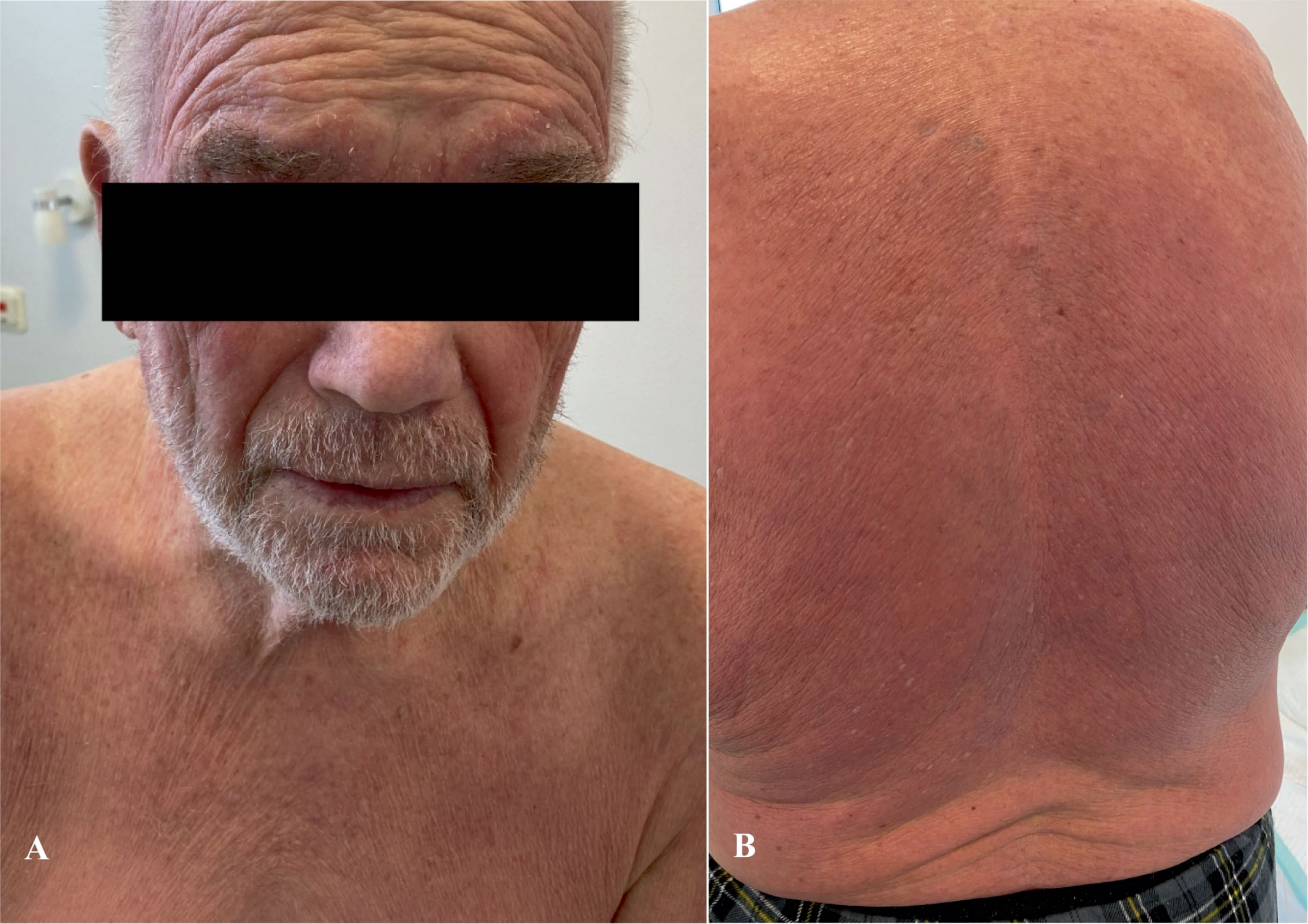
Clinical presentation demonstrates a significant improvement of erythroderma and hyperkeratotic crusts after 1 week of treatment: **(A)** face and **(B)** back.

The clinical, dermoscopic, and parasitological findings were consistent with a diagnosis of erythrodermic crusted scabies. Treatment with oral ivermectin (200 μg/kg on days 1, 2, 8, 9, and 15) in combination with topical 20% benzyl benzoate resulted in significant clinical improvement within 1 week, characterized by the resolution of scaling and a substantial reduction in erythroderma (Figure 3). To alleviate the associated inflammatory erythema, a high-potency topical corticosteroid was prescribed for short-term use until the erythema had substantially improved. Additionally, a topical preparation containing sulfur and 2% salicylic acid was used to address the hyperkeratotic component of the disease. The 20% benzyl benzoate was selected based on local availability and institutional practice. Although 5% permethrin is widely recommended as first-line topical therapy in many guidelines, benzyl benzoate remains an accepted alternative scabicidal agent, particularly in combination regimens for crusted scabies. In addition, the possibility of reduced efficacy or treatment failure with topical agents such as permethrin was considered in the context of severe crusted infestation with high mite burden and impaired drug penetration, supporting the use of combination therapy with oral ivermectin and an alternative topical scabicide.

## 4 Discussion

In our literature review, erythrodermic crusted scabies was most frequently misdiagnosed as erythrodermic psoriasis, likely due to overlapping clinical features, including crusting, hyperkeratosis, and scaling. Other reported initial diagnoses or differential considerations included erythroderma associated with hypothyroidism, hypereosinophilic syndrome, eczema, contact dermatitis, adverse drug reactions, cutaneous lymphoma, and other autoimmune conditions.

Diagnostic interpretation becomes increasingly complex when multiple dermatologic conditions coexist, such as a history of other dermatoses, including psoriasis, which is a known cause of erythroderma. Diagnostic challenges are heightened when less commonly affected areas, such as the face, are involved, as facial involvement is rare in crusted scabies (7). Additionally, facial and scalp involvement may mimic seborrheic dermatitis, which can also induce an erythrodermic state. These scenarios are particularly significant in primary care and general practice, where initial management decisions are made. This underscores the necessity of including scabies in the differential diagnosis of erythroderma presenting with pruritus and scaly or hyperkeratotic lesions before initiating immunosuppressive therapy. In this context, primary care plays a critical role in early recognition and maintaining diagnostic suspicion, especially given the rarity of erythrodermic crusted scabies and the limited number of cases reported in the literature. In our patient, erythrodermic psoriasis was initially considered among the leading differential diagnoses due to a history of psoriasis with prior elbow involvement. However, several clinical and diagnostic features were not consistent with erythrodermic psoriasis, including the presence of yellowish scales (whereas psoriasis is typically associated with silvery-white scaling), marked pruritus, dermoscopic findings suggestive of scabies, and the identification of *Sarcoptes scabiei* on skin scrapings. In addition, significant clinical improvement following anti-parasitic therapy supported an infectious etiology. Based on the overall clinical course and confirmatory parasitological findings, the final diagnosis was established as erythrodermic crusted scabies.

In our patient with a history of psoriasis, the psoriasiform histologic features may represent a Koebner phenomenon, defined as the development of lesions of a pre-existing dermatosis at sites of cutaneous injury, potentially triggered by scabies infestation (8). However, this mechanism remains speculative and may not account for all cases of erythroderma, even in patients with a history of psoriasis. Erythroderma in crusted scabies may also be exacerbated by *Staphylococcus aureus* colonization of scabies burrows (4). Scabies predisposes to secondary bacterial infection through disruption of the skin barrier, while mite-derived proteins, such as SMSB4, may inhibit complement activation and impair neutrophil bactericidal activity, thereby facilitating bacterial dissemination (4). Psoriasiform histopathological changes identified in scabies may result from alternative mechanisms such as chronic scratching, epidermal reactive alterations, or superimposed eczematization, rather than representing true psoriatic pathology. In this case, the histopathological findings were non-specific and required interpretation within the clinical context of erythroderma, as similar psoriasiform patterns may occur in a variety of inflammatory dermatoses. Accordingly, histopathology should not be used as a standalone diagnostic tool but rather interpreted in conjunction with clinical and parasitological findings. Greater diagnostic weight was therefore assigned to the positive skin scraping and dermoscopic findings, which confirmed the presence of *Sarcoptes scabiei*.

In our review, reported patient ages ranged from 3 to 90 years. Many individuals initially received immunosuppressive therapy, including corticosteroids, azathioprine, or mycophenolate mofetil, for presumed alternative diagnoses or underlying conditions. This treatment frequently resulted in clinical deterioration and contributed to the progression to erythroderma. Within our literature dataset, two elderly patients died from complications of their underlying diseases in association with sepsis. Furthermore, several patients exhibited risk factors such as neurologic or cognitive impairment, which may reduce cutaneous sensation or the perception of pruritus and impair the ability to mechanically remove mites (3).

The majority of cases were diagnosed using skin scrapings, either alone or combined with dermoscopy or histopathology, while a minority relied exclusively on biopsy. Available data indicate that skin scrapings and dermoscopy exhibit similar sensitivity, although skin scrapings have higher specificity (9). In clinical practice, particularly for scabies, these diagnostic modalities are most effective when used together, whereas histopathology should be reserved for cases that are diagnostically unclear. Skin scrapings remain a rapid and highly specific method for confirming or excluding scabies. This technique is also cost-effective and may be accessible in primary care settings, including those with limited resources. Other diagnostic approaches, such as dermoscopy and skin punch biopsy, may be used when appropriate and available, but their effectiveness also depends on the clinician’s expertise.

The therapeutic management of erythrodermic crusted scabies is similar to that of crusted scabies without erythroderma. Reported cases have used oral ivermectin in combination with topical scabicidal agents, including permethrin, benzyl benzoate, or sulfur preparations, although some patients have been managed with topical therapy alone. Based on these observations, a combination regimen of oral ivermectin and topical scabicide is appropriate for erythrodermic presentations. Ivermectin is typically administered at a dose of 200 μg/kg on multiple days (e.g., days 1, 2, 8, 9, 15 ± additional doses depending on severity and follow-up findings), while topical agents are generally applied in repeated courses, depending on the selected preparation (10).

The literature indicates ongoing debates regarding therapies for scabies. Concerns include the emergence of resistance to first-line treatments such as permethrin and systemic therapies (6). Due to concerns regarding the efficacy of permethrin, benzyl benzoate has, in some settings, been recommended as a first-line treatment option. It is important to distinguish true pharmacological resistance from so-called “pseudo-resistance,” which occurs when treatment failure results from incorrect application of topical therapies, poor adherence, or untreated close contacts (6). However, isolated cases of ivermectin resistance have been reported and may be associated with genetic mutations in Sarcoptes scabiei affecting drug targets or transport mechanisms (6). The literature also suggests that effective treatment may require more intensive regimens. Instead of two applications of 5% permethrin 1 week apart, daily applications or multiple weekly cycles are advised (6). Some guidelines recommend two doses of 200 μg/kg oral ivermectin, 1 week apart; however, because of its limited effect on ovids, multiple-dose regimens over a 7- to 14-day period may improve outcomes (6).

In addition to pharmacologic therapy, decontamination measures, including treating close contacts and appropriately laundering clothing, bedding, and towels, are essential to prevent reinfestation and recurrence (10). Given the risk of secondary bacterial infection and septic complications, antibiotic therapy should be guided by clinical evaluation, particularly in elderly or medically complex patients. In our case, stable vital signs and laboratory findings indicated that antibiotic therapy was not required. Adjunctive short courses of low-potency topical corticosteroids or low-dose systemic corticosteroids may be considered for symptomatic relief; however, these should not represent the mainstay of treatment. Post-treatment pruritus may persist for up to 4 weeks, and patient reassurance is therefore important (6). Symptomatic relief can be achieved with antihistamines and mild topical corticosteroids (10).

Scabies constitutes a significant global public health burden. Reducing its impact requires improved diagnostic accuracy, optimized therapeutic strategies, and robust public health interventions focused on prevention, early detection, and outbreak control. Ultraviolet dermoscopy demonstrates promising potential for detecting and visualizing burrows and mite structures by leveraging their fluorescence, which is often difficult to observe under conventional light (6). Reflective confocal microscopy enables imaging of scabies and its structures within the stratum corneum, while line-field confocal optical coherence tomography offers high-resolution cross-sectional visualization (6). Treatment options are expanding; for instance, moxidectin is currently under investigation as a systemic therapy for scabies (6). Additional therapies with improved safety profiles are being explored, including nanotechnology-based formulations and enzyme inhibitors that target mite-specific metabolic pathways (6). Research also emphasizes drug formulations that achieve high local efficacy without systemic involvement, such as high-concentration permethrin topical formulations and pediatric ivermectin use (6). A comprehensive public health response remains essential, integrating dermatologic care into primary care settings and prioritizing early diagnosis and treatment (6).

## 5 Conclusion

Crusted scabies should be considered in the differential diagnosis of erythroderma with hyperkeratosis, scaling, and pruritus, particularly in frail elderly patients with multiple comorbidities. The diagnosis should be reconsidered in cases of clinical deterioration during corticosteroid therapy initiated for an alternative presumed diagnosis. It is important to apply standardized diagnostic criteria when evaluating patients with suspected scabies, even in non-severe presentations, such as the 2020 International Alliance for the Control of Scabies consensus criteria. Early recognition of these cases is crucial, as progression to erythroderma represents a life-threatening condition, and crusted scabies further increases the risk of secondary bacterial infection and sepsis. In early disease stages, pruritus-driven scratching may facilitate the mechanical dissemination of mites, eggs, and fecal material to other skin sites, while also predisposing to secondary bacterial infection.

In primary care or non-dermatology settings, skin scrapings are recommended for erythrodermic patients, especially when hyperkeratotic plaques and scaling are present. Histopathology alone may be non-specific and, in some situations, insufficient as a standalone diagnostic tool without clinical and parasitological correlation.

Management of erythrodermic crusted scabies typically includes systemic ivermectin, dosed according to body weight, in combination with topical scabicidal agents. Adjunctive therapies, including antibiotics and, in selected cases, immunosuppressive treatment to control inflammation, may be considered with caution and close clinical monitoring. This study is limited by the small sample size and the possibility that some cases were not identified due to the database selection and search strategy.

This study has several limitations. As a case report, the findings are not generalizable to all patients with crusted scabies or erythroderma. Additionally, the histopathological examination did not reveal elements of scabies, which may be attributable to a non-representative biopsy site. Furthermore, the literature review was limited to selected databases and a specific search strategy, which may have resulted in the omission of relevant cases.

## Data Availability

This study is based on a single case report and a literature review; no original datasets were generated or analyzed. Any materials supporting the findings of this study are available from the corresponding author upon reasonable request at juris.med@mail.lv.

## References

[1] Harper-Kirksey K. (2018). Erythroderma. In: Rose, E. (eds) Life-Threatening Rashes. Cham: Springer. doi: 10.1007/978-3-319-75623-3_19

[2] Das A Bar C Patra A. Norwegian scabies: rare cause of erythroderma. Indian Dermatol Online J. (2015) 6:52–4. doi: 10.4103/2229-5178.148951, 25657922 PMC4314893

[3] Niode NJ Adji A Gazpers S Kandou RT Pandaleke H Trisnowati DM . Crusted scabies, a neglected tropical disease: case series and literature review. Infect Dis Rep. (2022) 14:479–91. doi: 10.3390/idr14030051, 35735761 PMC9223105

[4] Armega-Anghelescu A Closca RM Vlad DC Cioenaru FC Rakitovan M Cristodor P . Tiny troublemakers-a comprehensive approach to crusted scabies. Diagnostics. (2025) 15:680. doi: 10.3390/diagnostics15060680, 40150023 PMC11941280

[5] Laganà A Saia I Genovese G Visalli G D’Andrea G Sidoti S . Resurgence of scabies in Italy: the new life of an old disease. Parasite Epidemiol Control. (2024) 27:e00392. doi: 10.1016/j.parepi.2024.e00392 39628512 PMC11612372

[6] Tavoletti G Avallone G Sechi A Cinotti E Veraldi S Micali G . Scabies: an updated review from epidemiology to current controversies and future perspectives. Travel Med Infect Dis. (2025) 67:102878. doi: 10.1016/j.tmaid.2025.102878, 40754226

[7] Kalita IR Singh HV. Norwegian (crusted) scabies involving eyelids and conjunctiva. Indian J Paediatr Dermatol. (2021) 22:52–5. doi: 10.4103/ijpd.IJPD_61_20

[8] Liu JM Lin CY Chang FW Liu YP Liang CP Hsu RJ. Increased risk of psoriasis following scabies infection: a nationwide population-based matched cohort study. J Dermatol. (2018) 45:302–8. doi: 10.1111/1346-8138.14221, 29356052

[9] Shoukat Q Rizvi A Wahood W Coetzee S Wrench A. Sight the mite: a meta-analysis on the diagnosis of scabies. Cureus. (2023) 15:e34390. doi: 10.7759/cureus.34390, 36874720 PMC9976840

[10] Salavastru CM Chosidow O Boffa MJ Janier M Tiplica GS. European guideline for the management of scabies. J Eur Acad Dermatol Venereol. (2017) 31:1248–53. doi: 10.1111/jdv.14351, 28639722

[11] Binić I Janković A Jovanović D Ljubenović M. Crusted (Norwegian) scabies following systemic and topical corticosteroid therapy. J Korean Med Sci. (2010) 25:188–91. doi: 10.3346/jkms.2010.25.1.188, 20052371 PMC2800004

[12] Bollea Garlatti LA Torre AC Bollea Garlatti ML Galimberti RL Argenziano G. Dermoscopy aids the diagnosis of crusted scabies in an erythrodermic patient. J Am Acad Dermatol. (2015) 73:e93–5. doi: 10.1016/j.jaad.2015.04.061, 26282822

[13] Cartron AM Boettler M Chung C Trinidad JC. Crusted scabies in an elderly woman. Dermatol Online J. (2020) 26:13030/qt4c36775g. doi: 10.5070/d32610050466, 33147672

[14] Chen HJ Lu CY Huang GM Tang LL. Norwegian scabies presenting as erythroderma: a case report. World J Clin Cases. (2024) 12:4802–6. doi: 10.12998/wjcc.v12.i21.4802, 39070813 PMC11235473

[15] Cohen PR. Concurrent scabies incognito and crusted scabies with scalp lesions masquerading as erythrodermic dermatitis. J Drugs Dermatol. (2019) 18:105. 30681808

[16] Devi GC Hazarika N. Erythroderma secondary to crusted scabies. BMJ Case Rep. (2021) 14:e248000. doi: 10.1136/bcr-2021-248000, 34906960 PMC8671912

[17] Dragoš V Kecelj N Žgavec B. Crusted scabies in an 8-year-old child. Acta Dermatovenerol Alp Pannonica Adriat. (2004) 13:66–70.

[18] Fonseca V Price HN Jeffries M Alder SL Hansen RC. Crusted scabies misdiagnosed as erythrodermic psoriasis in a 3-year-old girl with down syndrome. Pediatr Dermatol. (2014) 31:753–4. doi: 10.1111/pde.12225, 24138478

[19] Kulkarni S Shah H Patel B Bhuptani N. Crusted scabies presenting as erythroderma in an HIV-seropositive patient. Indian J Sex Transm Dis AIDS. (2016) 37:72–4. doi: 10.4103/0253-7184.180285, 27190417 PMC4857688

[20] Olamiju B Leventhal JS Vesely MD. Crusted scabies presenting as erythroderma in a patient with immunosuppression. Cutis. (2023) 111:E44–7. doi: 10.12788/cutis.0794, 37406327

[21] Paparizos V Vasalou V Velissariou E . Norwegian scabies presenting as erythroderma in HIV: a case report. Infez Med. (2019) 27:332–5. 31545779

[22] Piana S Bernardi C Zoino JL. Itchy erythroderma in a neoplastic patient—mind the mite. Eur J Intern Med. (2015) 26:139–40. doi: 10.1016/j.ejim.2014.06.026, 25087690

[23] Ren YK Yuan HJ Sun W Lang XQ Liu HY Guo SP. Erythroderma-like crusted scabies in an immunocompetent patient after systemic corticosteroids. Skin Res Technol. (2024) 30:e13870. doi: 10.1111/srt.13870 39041654 PMC11264097

[24] Shrestha A Bischof E. Crusted scabies induced hypereosinophilic syndrome. Cureus. (2021) 13:e15201. doi: 10.7759/cureus.15201, 34178521 PMC8221644

[25] Talty R Micevic G Damsky W King BA. Erythrodermic scabies in an immunocompetent patient. JAAD Case Rep. (2022) 29:112–5. doi: 10.1016/j.jdcr.2022.08.045, 36262356 PMC9573821

[26] Widaty S Martinus M Rachmawati Y. Erythrodermic manifestation due to hyperinfestation of scabies. Berk Ilmu Kesehat Kulit Kelamin. (2021) 33:141–4. doi: 10.20473/bikk.V33.2.2021.141-144

