# Supplementary figures and images for: Erythrodermic crusted scabies—a diagnostic pitfall and mini-review: a case report
Source: Front Med (Lausanne). 2026 Jul 13;13:1862964. doi: 10.3389/fmed.2026.1862964 (PMC13402148; doi:10.3389/fmed.2026.1862964)

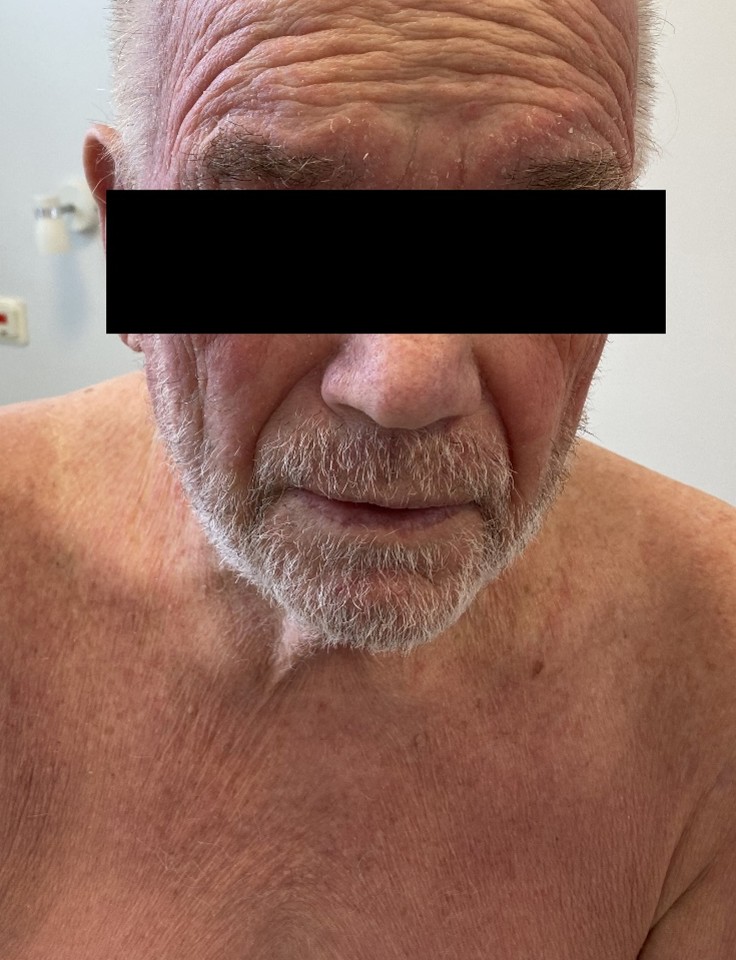

Supplement: Supplementary file 1 [file Image_1.JPEG]

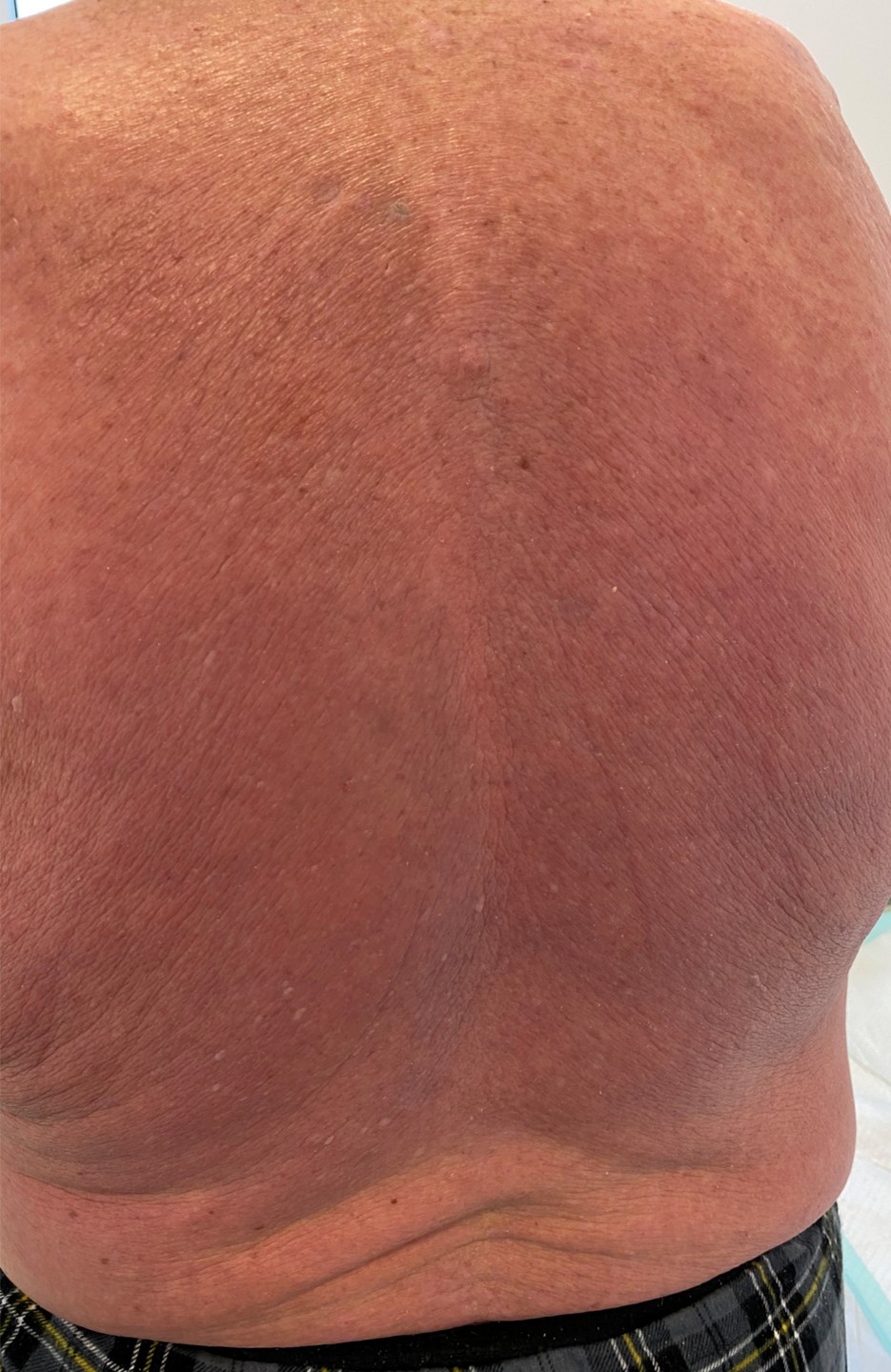

Supplement: Supplementary file 2 [file Image_2.JPEG]
